# Author Correction: The rhythmic coupling of Egr-1 and Cidea regulates age-related metabolic dysfunction in the liver of male mice

**DOI:** 10.1038/s41467-026-75641-1

**Published:** 2026-07-14

**Authors:** Jing Wu, Dandan Bu, Haiquan Wang, Di Shen, Danyang Chong, Tongyu Zhang, Weiwei Tao, Mengfei Zhao, Yue Zhao, Lei Fang, Peng Li, Bin Xue, Chao-Jun Li

**Affiliations:** 1 Ministry of Education Key Laboratory of Model Animal for Disease Study, Model Animal Research Center of the Medical School, Nanjing University, Nanjing, 210093 Jiangsu Province China; 2 State Key Laboratory of Reproductive Medicine and China International Joint Research Center on Environment and Human Health, Center for Global Health, School of Public Health, Nanjing Medical University, Nanjing, 211166 China; 3 Core Laboratory, Sir Run Run Hospital, Nanjing Medical University, Nanjing, 211166 Jiangsu China; 4 Institute of Metabolism & Integrative Biology (IMIB), Fudan University, Shanghai, 200438 China

**Keywords:** Circadian rhythms, Gene regulation, Ageing, Fat metabolism

Correction to: *Nature Communications* 10.1038/s41467-023-36775-8, published online 24 March 2023

In the version of this article initially published, due to a figure preparation error, the representative WT panel in Fig. 3b was a duplicate of the H&E panel shown in Fig. 1b. Figure 3 has now been updated in the HTML and PDF versions of the article.

