# Supplementary material for: The rhythmic coupling of Egr-1 and Cidea regulates age-related metabolic dysfunction in the liver of male mice
Source: Nat Commun. 2023 Mar 24;14:1634. doi: 10.1038/s41467-023-36775-8 (PMC10038990; doi:10.1038/s41467-023-36775-8)
Supplement: Supplementary file 1 — Supplementary information [file 41467_2023_36775_MOESM1_ESM.pdf]

The rhythmic coupling of Egr-1 and Cidea regulates age-related metabolic dysfunction in the liver of male mice

Jing Wu[1,2,3#], Dandan Bu[1#], Haiquan Wang[1], Di Shen[1], Danyang Chong[1], Tongyu Zhang[1], Weiwei Tao[2], Mengfei Zhao[1], Yue Zhao[1], Lei Fang[1], Peng Li[4*], Bin Xue[3*] and Chao-Jun Li[2*]

Supplementary Figures

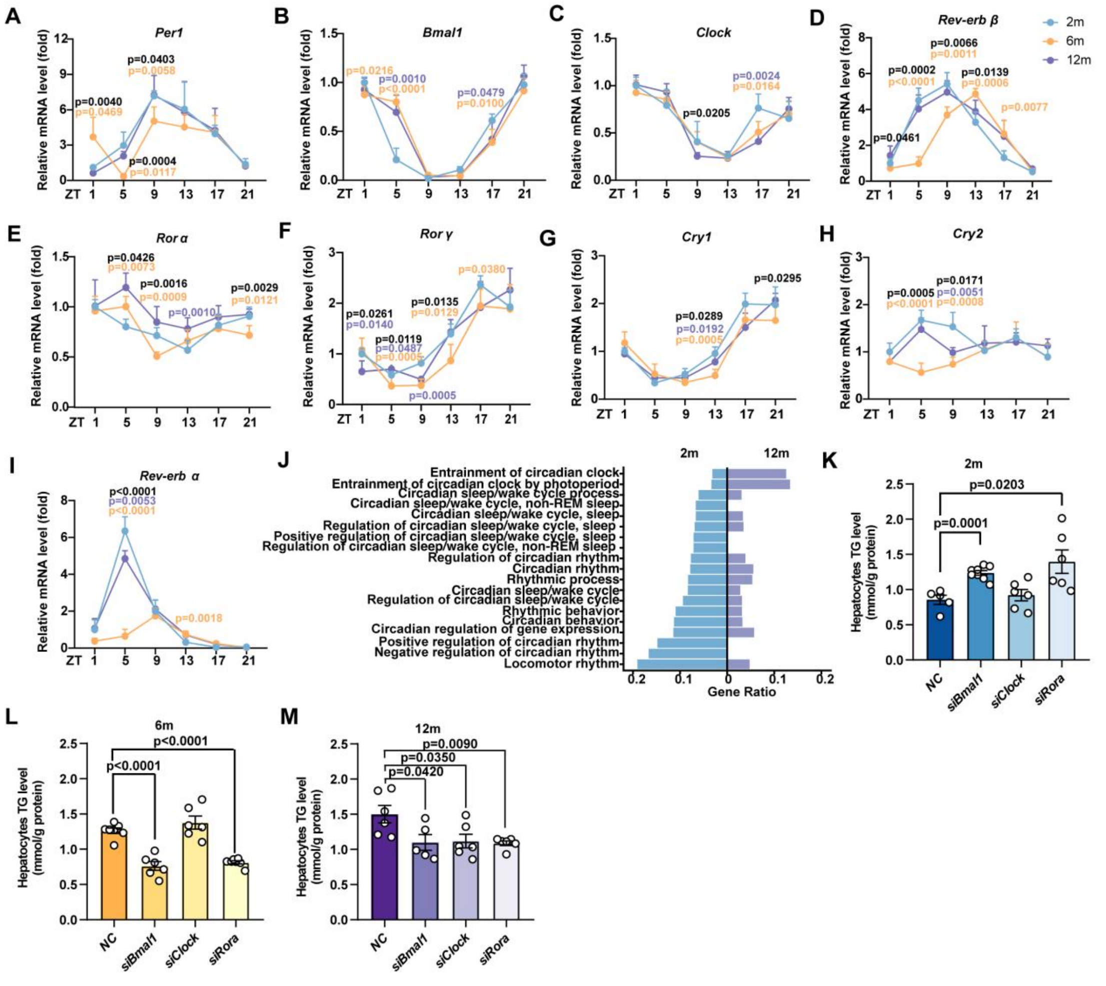

Supplementary Figure 1. The liver circadian system regulation on lipid metabolism is shifted with aging.

A-I.mRNA level of the clock genes and clock-controlled genes at the indicated time points in the livers of C57BL/6 mice at 2 months, 6 months, and 12 months (n=5 biologically independent animals per group); J. Selected significantly enriched circadian rhythm related GO terms of 2-month and 12-month intersect genes; K-M. TG levels in WT primary hepatocytes from 2-month-old, 6-month-old and 12-month-old mice after infection with a siRNA by

knocking down Bmal1 or Clock or Rora (2month: *NC*:n=5; *SiBmal1*: n-8; *SiClock*: n=6; *SiRora*: n=6; 6month: *NC*:n=7; *SiBmal1*: n-6; *SiClock*: n=6; *SiRora*: n=7; 12month: *NC*:n=6; *SiBmal1*: n-5; *SiClock*: n=6; *SiRora*: n=6 biologically independent samples). Each experiment was repeated three times independently. Data are represented as mean ± SEM. Exact p-values are depicted in the figure. Orange color p-value means 6month versus 2month group; Purple color p-value means 12month versus 2month group; Black color p-value means 6month versus 12month group. Statistical analysis was performed using One-way ANOVA. Source data are provided as a Source Data file.

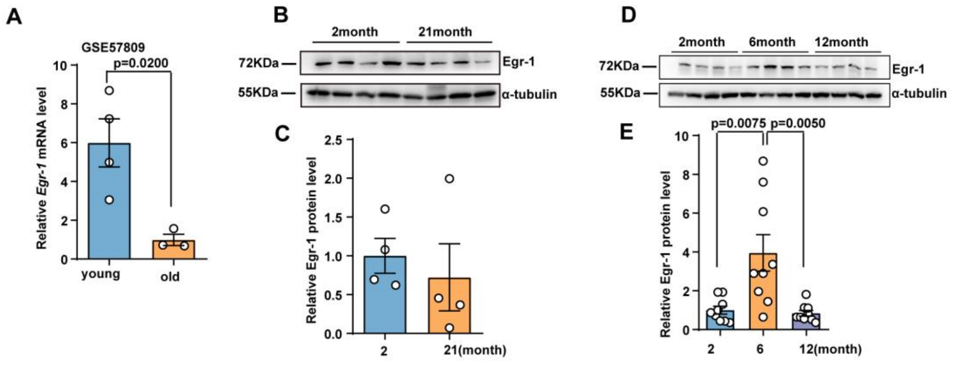

Supplementary Figure 2. The phase of the Egr-1 circadian rhythm in the liver moves forward with aging.

A. mRNA levels of Egr-1 in young and old mice according to gene array analysis (GSE57809). B-C. Immunoblot and quantitative analysis of Egr-1 expression levels in the livers of C57BL/6 mice at 2 months and 21 months (n=4 biologically independent animals per group);

D-E. Protein expression and quantitative analysis of Egr-1 in the livers of C57BL/6 mice at 2 months, 6 months and 12 months (n=9 biologically independent animals per group). Data are represented as mean ± SEM. *P < 0.05, unpaired t test. Data are represented as mean ± SEM. Exact p-values are depicted in the figure. Statistical analysis was performed using unpaired two-tailed Student’s t-test for A, C and One-way ANOVA for E. Source data are provided as a Source Data file.

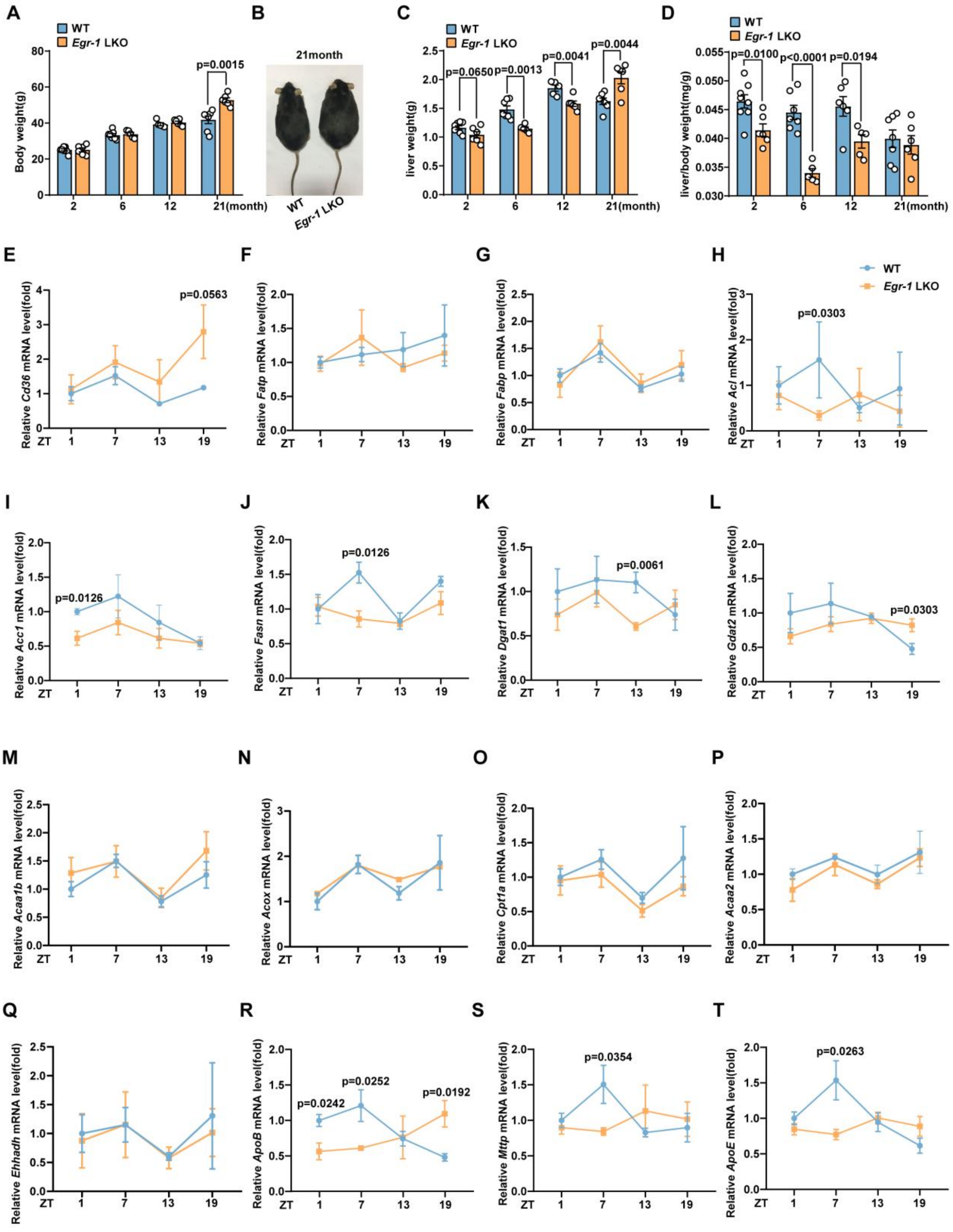

Supplementary Figure 3. *Egr-1* deficiency facilitates fatty acid uptake to enable accumulation of excessive fatty acids from 6 months onward.

WT and *Egr-1*-LKO mice at 2 months, 6 months, 12 months and 21 months were examined (WT: 2month: n=8; 6month: n=7; 12month: n=5; 21month: n=7; *Egr-1* LKO: 2month: n=6;

6month: n=5; 12month: n=6; 21month: n=6 biologically independent animals). A. Body weight. B. Photographs of WT and *Egr-1*-LKO mice at 21 months of age. C. Liver weight. D. Liver weight/body weight ratio. mRNA expression of genes at the indicated time points related to fatty acid uptake(E-G), fatty acid synthesis(H-L), fatty acid oxidation(M-Q) and TG transport (R-T) in the livers of WT and *Egr-1*-LKO mice at 6 months of age (n=4 biologically independent animals per group). Data are represented as mean ± SEM. Exact p-values are depicted in the figure. Statistical analysis was performed using One-way ANOVA. Source data are provided as a Source Data file.

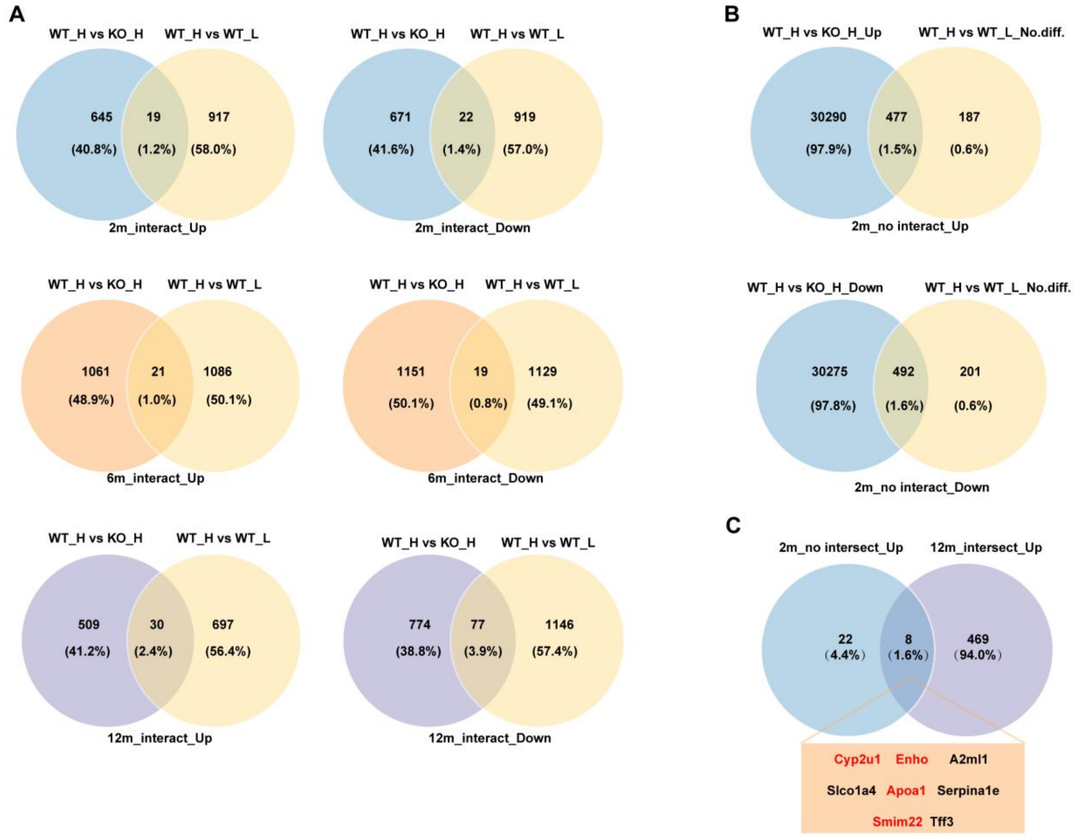

Supplementary Figure 4. Transcriptomic analysis of the liver in *Egr-1*-deleted mice with age increased.

A. Venn diagram represents the overlap among the indicated groups. B. Venn diagram represents the overlap between WT_H vs KO_H_up/down and WT_H vs WT_L_no. diff. group; C.Venn diagrams representing the overlap between 2m_no_intersect_up and 12_intersect_up group. The lipid metabolism related genes were labeled with red font.

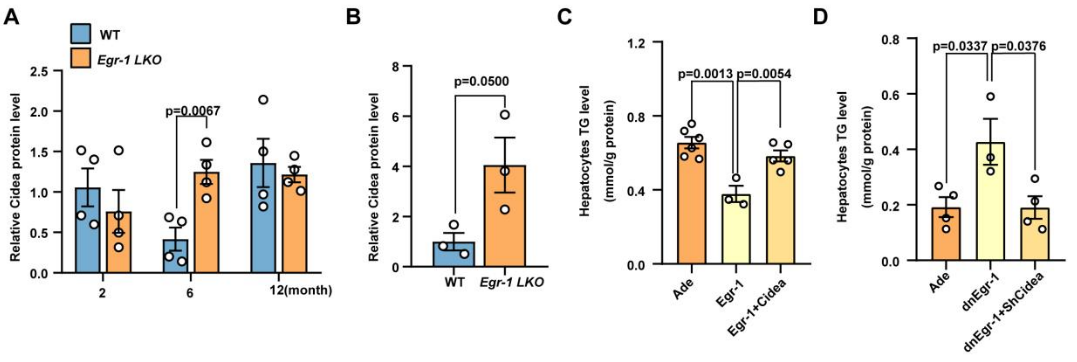

Supplementary Figure 5. Egr-1 regulates liver metabolic aging in a Cidea-dependent manner. A. Quantitative analysis of Cidea protein levels in Figure 5B (n = 4 biologically independent animals per group). B. Quantitative analysis of Cidea protein levels in Figure 5C (n=3 biologically independent experiments). C. Hepatocyte TG levels after transfection with a Cidea overexpression plasmid or infection with an Egr-1 overexpression adenovirus, which cell cultured in DMEM without glucose but with 10% FBS (Ade: n= 6; Egr-1: n=3; Egr-1+Cidea: n=5 biologically independent samples); D. TG levels in primary hepatocytes from 6-month-old mice after infection with a ShCidea adenovirus or infection with an dnEgr-1 overexpression adenovirus, which cell cultured in DMEM without glucose but with 10% FBS (Ade: n= 4; dnEgr-1: n=3; dnEgr-1+ShCidea: n=4 biologically independent samples). Each experiment was repeated three times independently. Data are represented as mean ± SEM. Exact p-values are depicted in the figure. Statistical analysis was performed using unpaired two-tailed Student’s t-test for B and One-way ANOVA for A, C, D. Source data are provided as a Source Data file.

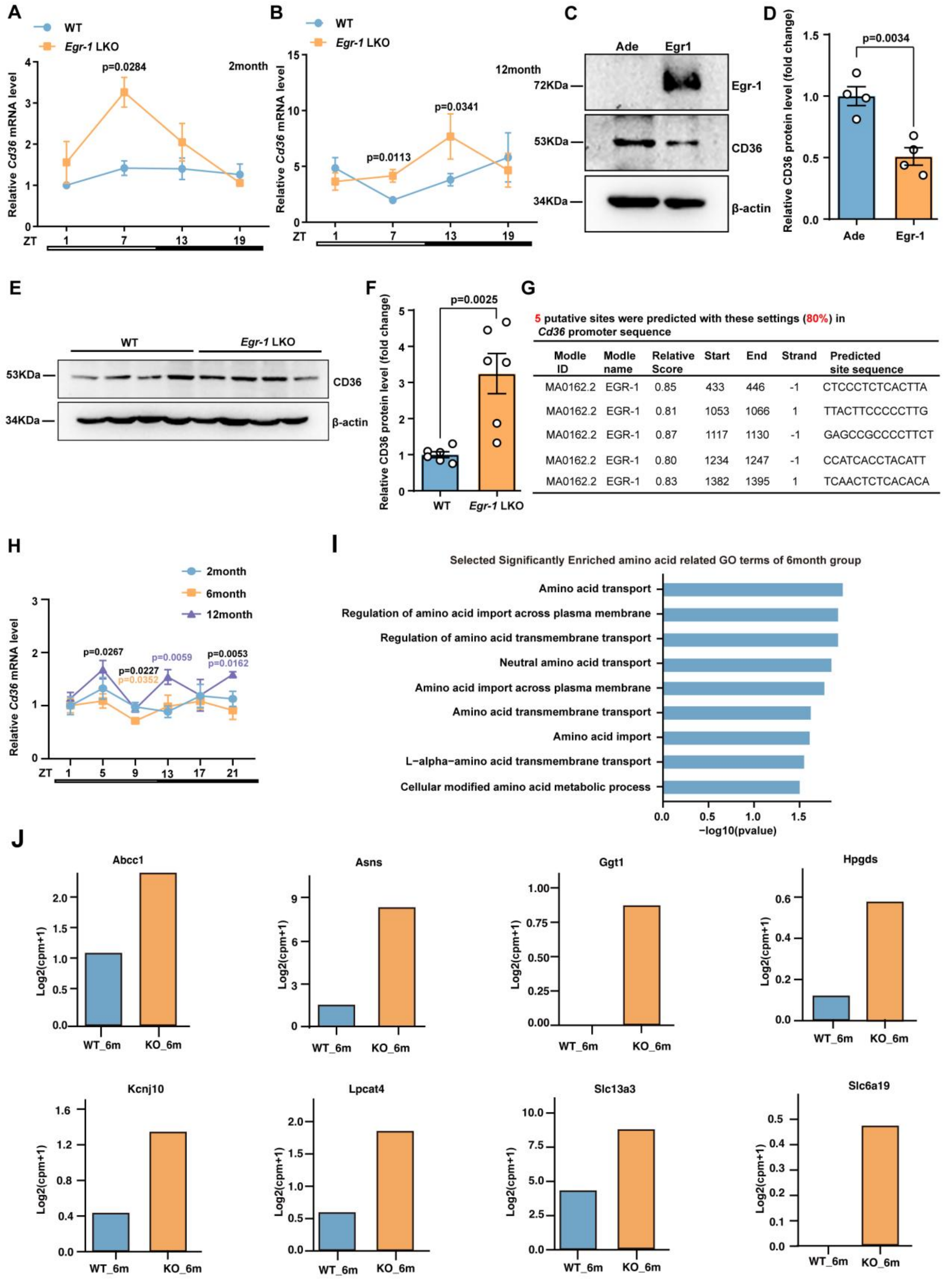

Supplementary Figure 6. ***Egr-1*** **deletion can enhance CD36 expression.**

A. the mRNA level of *Cd36* at the indicated time points at 2-month-old mice (WT ZT1-19: n=3; *Egr-1* LKO ZT1,13,19: n=3, ZT7: n=4 biologically independent animals). B. the mRNA level of *Cd36* at the indicated time points at 12-month-old mice (WT ZT1,7,19: n=3;

ZT13:n=8; *Egr-1* LKO ZT1,7,13: n=4, ZT19: n=3 biologically independent animals). C-D. protein level and quantitative analysis of CD36 when overexpressing Egr-1 (n=4 independently experiments). E-F. protein level and quantitative analysis of CD36 in WT and *Egr-1* LKO mice (n=6 biologically independent animals per group). G. five putative Egr-1 binding sites in Cd36 promoter sequence. H. mRNA of the CD36 at the indicated time points in the livers of C57BL/6 mice at 2 months, 6 months, and 12 months (n=5 biologically independent animals per group). I. selected significantly Enriched amino acid related GO terms of changed genes between WT and Egr-1 LKO (KO) group at 6-month-old; J. significantly changed genes in amino acid related GO terms in I. Data are represented as mean ± SEM. Exact p-values are depicted in the figure. Orange color p-value means 6month versus 2month group; Purple color p-value means 12month versus 2month group; Black color p-value means 6month versus 12month group. Statistical analysis was performed using unpaired two-tailed Student's t-test for D, F and One-way ANOVA for A, B, H. Source data are provided as a Source Data file.

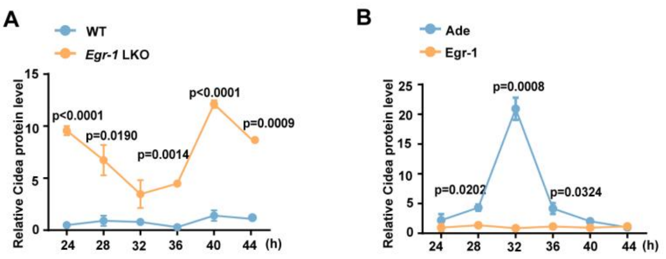

Supplementary Figure 7. Egr-1/BMAL1/CLOCK regulates the robustness and rhythm of Cidea by inhibiting its transcription.

A-B. Quantitative analysis of Cidea protein levels in Figure 6E-F. n=3 independently experiments. Data are represented as mean ± SEM. Exact p-values are depicted in the figure. Statistical analysis was performed using One-way ANOVA. Source data are provided as a Source Data file.

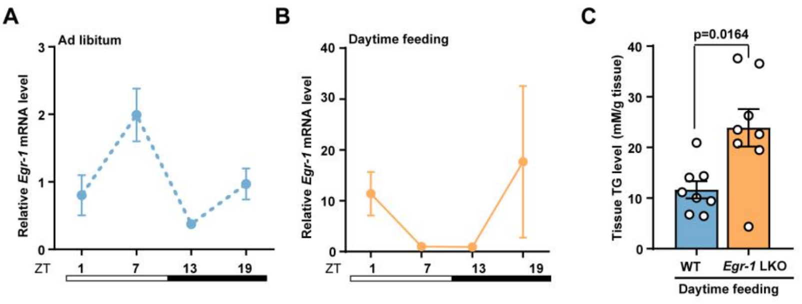

Supplementary Figure 8. Restriction of feeding to daytime does not reduce TG accumulation.

A-B. mRNA levels of *Egr-1* in ad libitum-fed and day-fed mice (n=4 biologically independent animals per group). C. Liver TG levels of WT and *Egr-1*-LKO mice restricted to daytime feeding (WT: n=7; *Egr-1* LKO: n=9 biologically independent animals). Data are represented as mean ± SEM. Exact p-values are depicted in the figure. Statistical analysis was performed using unpaired two-tailed Student's t-test. Source data are provided as a Source Data file.

Supplementary Table 1. Sequence of Primers.

Gene	Sequence (3')
Cre	TGCCACGACCAAGTGACAGCAATG
	AGAGACGGAAATCCATCGCTCG
Egr-1-Loxp	CCTTTCCTCACTCACCCACCATGG
	CACCCACGCAGCTTGAGTTCTC
36B4(mouse)	GAAACTGCTGCCTCACATCCG
	GCTGGCACAGTGACCTCACACG
Egr-1(mouse)	GTCCTTTTCTGACATCGCTCTGA
	CGAGTCGTTTGGCTGGGATA
α-SMA	GTCCCAGACATCAGGGAGTAA
	TCGGATACTTCAGCGTCAGGA
Cidea(mouse)	TGACATTCATGGGATTGCAGAC
	GGCCAGTTGTGATGACTAAGAC
Clock	CACTCTCACAGCCCCACTGTAC

	CCCCACAAGCTACAGGAGCAGT
Bmal1	TGGAGGGACTCCAGACATTC
	TGGGACTACTTGATCCTTGG
Rev-erbα	TGCAGGCTGATTCTTCACACA
	AGCCCTCCAGAAGGGTAGGA
Rev-erbβ	CGCACATTGCCGATATAGGAGG
	GAGACTGCCACCACCACGTACT
Rorα	CCAACCGTGTCCATGGCAGAAC
	GCACACAGCTGCCACATCACCT
Rorγ	GGCAGCGCACCAACCTCTTTTC
	CTGGTCATTCTGGCAGAGCTCC
Cry1	AGCGCAGGTGTCGGTTATGAGC
	ATAGACGCAGCGGATGGTGTCG
Cry2	TGGGCATCAACCGATGGAG
	CCCATTCCTTGAACAGCCTTG
Per1	AACGGGATGTGTTTCGGGGTGC
	AGGACCTCCTCTGATTCGGCAG
Per2	TGATCGAGACGCCTGTGCTCGT
	CTCCACGGGTTGATGAAGCTGG
mCD36	TTGTACCTATACTGTGGCTAAATGAGA
	CTTGTGTTTTGAACATTTCTGCTT
mFATP	CACGATCCCGTGCATCTTCC
	AGCATTGGAGTAGGTGTCCAG
mFABP	GTGGAAAGTAGACCGGAACGA
	CCATCCTGTGTGATTGTCAGTT
mAcaa1b	ATGCTTCCATGCTGAGATTGT
	TCCATCCTTGAAGGCAGGCTT
mAcox1	GCCTGCTGTGTGGGTATGTCATT
	GTCATGGGCGGGTGCAT
mEhhadh	GTGATTGGCACCCACTTCTT
	TCGATTCCCAACAAATCCAT
mEch1	AAGATAAGGACGCCATGCTGAA
	TCCAGGTGGCCATGTAGTCA
mCpt1a	CTCAGTGGGAGCGACTCTTCA

	GGCCTCTGTGGTACACGACAA
Acaa2	CTGCTACGAGGTGTGTTCATC
	AGCTCTGCATGACATTGCCC
mHadha	GTGTTTGAGGACCTCGGTGT
	CGTTGTGTCCTTGGAGGTTT
Acadl	TCTTTTCCTCGGAGCATGACA
	GACCTCTCTACTCACTTCTCCAG
Acadvl	CTACTGTGCTTCAGGGACAAC
	CAAAGGACTTCGATTCTGCCC
mACL	GCCAGCGGGAGCACATC
	CTTTGCAGGTGCCACTTCATC
ACC1	ATGGGCGGAATGGTCTCTTTC
	TGGGGACCTTGTCTTCATCAT
FAS	CTGCGATTCTCCTGGCTGTGAA
	CAACAACCATAGGCGATTTCTGG
ELVOL6	CCCGAACTAGGTGACACGAT
	CCAGCGACCATGTCT TTGTA
SCD1	ATGTCTGACCTGAAAGCCGA
	GAAGGTGCTAACGAACAGGC
Gpat1	ACAGTTGGCACAATAGACGTTT
	CCTTCCATTTCAGTGTTGCAGA
Gpat2	CACTGCTCCGAGGTTTTGATG
	AGGTTGGCAGCAATTCCATAC
Dgat1	TCCGTCCAGGGTGGTAGTG
	TGAACAAAGAATCTTGCAGACGA
Dgat2	GCGCTACTTCCGAGACTACTT
	GGGCCTTATGCCAGGAAACT
mApoB	TTGGCAAACTGCATAGCATCC
	TCAAATTGGGACTCTCCTTTAGC
MTTP	CAGGGTGGTCTAGCTATTGAT
	TTTCACTCGGGTTTTAGACTC
mApoE	CTCCCAAGTCACACAAGAACTG
	CCAGCTCCTTTTTGTAAGCCTTT
