# Supplementary material for: The rhythmic coupling of Egr-1 and Cidea regulates age-related metabolic dysfunction in the liver of male mice
Source: Nat Commun. 2023 Mar 24;14:1634. doi: 10.1038/s41467-023-36775-8 (PMC10038990; doi:10.1038/s41467-023-36775-8)
Supplement: Supplementary file 3 — Reporting Summary [file 41467_2023_36775_MOESM3_ESM.pdf]

Corresponding author(s): Chaojun Li
Last updated by author(s): 01/19, 2023

Reporting Summary

Nature Portfolio wishes to improve the reproducibility of the work that we publish. This form provides structure for consistency and transparency in reporting. For further information on Nature Portfolio policies, see our Editorial Policies and the Editorial Policy Checklist.

Statistics

For all statistical analyses, confirm that the following items are present in the figure legend, table legend, main text, or Methods section.

n/a	Confirmed	
☐	☒	The exact sample size (*n*) for each experimental group/condition, given as a discrete number and unit of measurement
☐	☒	A statement on whether measurements were taken from distinct samples or whether the same sample was measured repeatedly
☐	☒	The statistical test(s) used AND whether they are one- or two-sided<br>*Only common tests should be described solely by name; describe more complex techniques in the Methods section.*
☐	☒	A description of all covariates tested
☐	☒	A description of any assumptions or corrections, such as tests of normality and adjustment for multiple comparisons
☐	☒	A full description of the statistical parameters including central tendency (e.g. means) or other basic estimates (e.g. regression coefficient) AND variation (e.g. standard deviation) or associated estimates of uncertainty (e.g. confidence intervals)
☐	☒	For null hypothesis testing, the test statistic (e.g. *F*, *t*, *r*) with confidence intervals, effect sizes, degrees of freedom and *P* value noted<br>*Give P values as exact values whenever suitable.*
☒	☐	For Bayesian analysis, information on the choice of priors and Markov chain Monte Carlo settings
☒	☐	For hierarchical and complex designs, identification of the appropriate level for tests and full reporting of outcomes
☒	☐	Estimates of effect sizes (e.g. Cohen's *d*, Pearson's *r*), indicating how they were calculated

Our web collection on statistics for biologists contains articles on many of the points above.

Software and code

Policy information about availability of computer code

Data collection	Immunofluorescence microscopy images were acquired using Olympus SpinSR microscope. Western blot data was acquired digitally by Image LAB(Bio-Red) software and analyzed by . Q-PCR data was acquired using the Applied Biosystems Viia 7 fast Real Time PCR system software.
Data analysis	Data were analyzed using Prism Graphpad version 8. Microscopy images analysis was performed using ImageJ software version 2.1.0/1.53c. Densitometric analysis of western blots was performed with ImageJ software version 2.1.0/1.53c. RNA-seq data were analyzed using edgeR software version 3.38.1.

For manuscripts utilizing custom algorithms or software that are central to the research but not yet described in published literature, software must be made available to editors and reviewers. We strongly encourage code deposition in a community repository (e.g. GitHub). See the Nature Portfolio guidelines for submitting code & software for further information.

Data

Policy information about availability of data

All manuscripts must include a data availability statement. This statement should provide the following information, where applicable:

- Accession codes, unique identifiers, or web links for publicly available datasets
- A description of any restrictions on data availability
- For clinical datasets or third party data, please ensure that the statement adheres to our policy

All transcriptome sequencing data that support the findings of this research have been deposited in the Gene Expression Omnibus (GEO) and are accessible through

the GEO accession number GSE195456 (https://www.ncbi.nlm.nih.gov/geo/query/acc.cgi?acc=%20GSE195456). All other data generated or analysed during this study are included in this published article (and its supplementary information files). Source data are provided with this paper.

Human research participants

Policy information about studies involving human research participants and Sex and Gender in Research.

Reporting on sex and gender	non
Population characteristics	*Describe the covariate-relevant population characteristics of the human research participants (e.g. age, genotypic information, past and current diagnosis and treatment categories). If you filled out the behavioural & social sciences study design questions and have nothing to add here, write "See above."*
Recruitment	*Describe how participants were recruited. Outline any potential self-selection bias or other biases that may be present and how these are likely to impact results.*
Ethics oversight	*Identify the organization(s) that approved the study protocol.*

Note that full information on the approval of the study protocol must also be provided in the manuscript.

Field-specific reporting

Please select the one below that is the best fit for your research. If you are not sure, read the appropriate sections before making your selection.

☒ Life sciences ☐ Behavioural & social sciences ☐ Ecological, evolutionary & environmental sciences

For a reference copy of the document with all sections, see nature.com/documents/nr-reporting-summary-flat.pdf

Life sciences study design

All studies must disclose on these points even when the disclosure is negative.

Sample size	No statistical methods were used to predetermine sample size(n). Number of sample was determined based on experimental approach, availability, feasibility required to obtain definitive results.
Data exclusions	Exclusion criteria for animals were applied in case of death, cannibalism and the presence of severe clinical alteration of vita physiological functions. Exclusion criteria for samples were applied in case of histological artifacts (freeze- and cut-damaged tissues), RNA and protein degradation.
Replication	All of experiments have been successfully repeated at least three times and/or with sufficient cells/animals per group to demonstrate statistical significance. All experiments were statistically analyzed.
Randomization	The mice were randomly assigned to each experimental/contrl group.
Blinding	Each mouse was assigned a code number to enable blinded experimental/control group; when applicable, experimenters were blinded to the nature of samples by using number codes until final data analysis was performed.

Reporting for specific materials, systems and methods

We require information from authors about some types of materials, experimental systems and methods used in many studies. Here, indicate whether each material, system or method listed is relevant to your study. If you are not sure if a list item applies to your research, read the appropriate section before selecting a response.

Materials & experimental systems

n/a	Involved in the study	
☐	☒	Antibodies
☐	☒	Eukaryotic cell lines
☒	☐	Palaeontology and archaeology
☐	☒	Animals and other organisms
☒	☐	Clinical data
☒	☐	Dual use research of concern

Methods

n/a	Involved in the study	
☒	☐	ChIP-seq
☒	☐	Flow cytometry
☒	☐	MRI-based neuroimaging

Antibodies

Antibodies used

Anti-Egr-1 (S-25) mouse monoclonal antibody , Santa Cruz , Cat #sc-101033,Western blot(WB)(1:500)
Anti-Cidea rabbit polyclonal antibody, Abcam, Cat #ab8402,Western blot(WB)(1:1000)
Anti-CD36(SMф) mouse monoclonal antibody , Santa Cruz , Cat #sc-7309,Western blot(WB)(1:1000)
Anti-CLOCK rabbit polyclonal antibody , Proteintech, Cat # 18094-1-AP, Western Blot (WB)(1:1000)
Anti-c-Myc rabbit polyclonal antibody , Proteintech, Cat # 10828-1-AP, Western Blot (WB)(1:1000)
Anti-β-actin mouse monoclonal antibody , Proteintech, Cat # 66009-1-Ig, Western Blot (WB)(1:2000)
Anti-α-tubulin?clone 1E4C11? mouse monoclonal antibody,proteintech,Cat # 66031-1-lg,WB?1:2000?

Validation

Anti-Egr-1 (S-25) (Santa Cruz,sc-101033) was validates by Western Blot(WB) analyses of extracts from Raw 264.7,and Immunofluorescence analysis(IF) of HeLa cells. The antibody was also validated in our lab by WB in primary hepatocytes.
Anti-Cidea (Abcam, ab8402)as validates by Western Blot(WB) analyses of extracts from Murine heart tissue lysate,and Immunofluorescence analysis(IF) of Mouse Heart cells.The antibody was also validated in our lab by WB in primary hepatocytes.
Anti-CD36(SMф) (Santa Cruz,sc-7309) was validates by Western Blot(WB) analyses of extracts from HUV-EC-C ,and Immunohistochemical analysis(IHC) of paraffin-embedded human heart muscle tissue. The antibody was also validated in our lab by WB in primary hepatocytes, with or without overexpression Egr-1 adenovirus.
Anti-CLOCK(Proteintech,18094-1-AP) was validates by Western Blot(WB) analyses of extracts from HEK-293 cells, HeLa cells, PC-3 cells,and Immunofluorescence analysis(IF) of HeLa cells. The antibody was also validated in our lab by WB in HEK293T cells.
Anti-c-Myc (Proteintech?10828-1-AP) was validates by Western Blot(WB) analyses of extracts from HeLa cells, HT-29 cells, HL-60 cells, HepG2 cells, human placenta tissue, A549 cells, Jurkat cells, Raji cells, SH-SY5Y cells, Jurkat cells and Raji cells, Immunoprecipitation analysis(IP) of MCF-7 cells, Immunofluorescence analysis(IF) of U2OS cells, HEK-293 cells and Flow cytometric (FC)analvsis of HeLa cells. The antibody was also validated in our lab by WB in HEK293T cells.
Anti-β-actin(Proteintech?66009-1-Ig) was validates by Western Blot(WB) analyses of extracts from HeLa cells, A549 cells, Jurkat cells, HSC-T6 cells. NIH/3T3 cells, Pig brain, Rabbit brain, Rat brain, Mouse brain, Chicken brain, HEK-293 cells, HepG2 cells, K-562 cells, HHSC-T6 cells, 4T1 cells, CHO cells, mouse pancreas, rat pancreas, Immunoprecipitation analysis(IP) of HeLa cells, Immunohistochemistry analysis(IHC) of human kidney tissue, human brain tissue, human colon cancer tissue, human heart tissue, Immunofluorescence analysis(IF) of MDCK cells, HeLa cells and Flow cytometric (FC)analvsis of HeLa cells.
Anti-α-tubulin (Proteintech, 66031-1-Ig) was validates by Western Blot(WB) analyses of extracts from HeLa cells, Recombinant protein.

Eukaryotic cell lines

Policy information about cell lines and Sex and Gender in Research

Cell line source(s)

HEK293T cell line are originated from ATCC(CRL-3216™).

Authentication

The cell line used was extracted DNA for comparison in DNA database for authentication.

Mycoplasma contamination

HEK 293T cell line wad maintained under the recommended culture conditions and media requirements. Mycoplasma detection was performed in accordance with department protocols (and tested negative)

Commonly misidentified lines
(See ICLAC register)

None

Animals and other research organisms

Policy information about studies involving animals; ARRIVE guidelines recommended for reporting animal research, and Sex and Gender in Research

Laboratory animals

We generated mice with liver-specific KO (LKO) of Egr-1 by crossing Alb-Cre transgenic mice with homozygous floxed Egr-1 mice. Littermates were used as controls. The KO lines (strain 129) were backcrossed for a minimum of six generations to the C57BL/6J background (Egr-1-loxp mouse background). The mice were housed in a controlled environment with a 12-hour/12-hour light/dark cycle at 20~24?and 50~65% relative humidity and were fed chow diet ad libitum. Chow diet for reproduction and maintenance of mice were from Xietong Shengwu, China (Reproduction: SFS9112; Maintenance: SWS9102).All the animals used in the study were male mice at 2 months, 6 months, 12 months, and 21 months of age. To analyze the lipid metabolism in Figure 3, the liver were dissected at Egr-1 highest(H) zeitgeber time in the male mice of different ages. Liver samples were obtained at ZT13(H) in 2month group; at ZT9(H) in 6month group; at ZT5(H) in 12month group and 21month group. To analyze genes rhythm expression, the livers of WT and Egr-1 LKO male mice at matched age were obtained every 6 h started at ZT1. To analyze the function of Egr-1 in age-related liver lipid accumulation, the light time for 6-month-old male mice was advanced by 4 hours for 1 month according to the time at which the peak of Egr-1 expression moved forward from ZT13 to ZT9. The livers of male mice were dissected at ZT5 for analyzing the lipid metabolism. For restricted feeding, the WT and Egr-1 LKO male mice for 6-month-old mice were fed exclusively at daytime for 1 month. The livers of male mice were dissected every 6 h started at ZT1 for analyzing the rhythm. Mouse liver samples were obtained by cervical dislocation after anesthesia with or without light and the mouse carcasses were treated with centralized pollution-free treatment.In the survival experiment, the animals were euthanized when animals in the state of no anesthesia or sedation, were unable to eat or drink, and stand or extremely reluctantly to stand up to 24 hours. The animals were monitored once a week and once a day if symptom described above were present. Moribund animals were euthanized and every animal found dead or euthanized was necropsied. The criteria for euthanasia were based on an independent assessment by the veterinarian according to the AAALAC guidelines, and the animal was represented as dead in the curve only once its condition was deemed unsuitable for continued survival. Animals in the survival curve (WT group: n=22; Egr-1 LKO group: n=23) were considered as censored deaths.

Wild animals	The study did not involved in wild animals.
Reporting on sex	male mice
Field-collected samples	The study did not involved in samples collected from the field.
Ethics oversight	All mice were maintained and used in accordance with the Animal Care and Use Committee of the Model Animal Research Center of Nanjing University, Nanjing, China, using approved protocols from the institutional animal care committee (#CS20).

Note that full information on the approval of the study protocol must also be provided in the manuscript.
